# Whole genome sequencing and comparative genomic analyses of two *Vibrio cholerae* O139 Bengal-specific *Podoviruses* to other N4-like phages reveal extensive genetic diversity

**DOI:** 10.1186/1743-422X-10-165

**Published:** 2013-05-28

**Authors:** Derrick E Fouts, Jochen Klumpp, Kimberly A Bishop-Lilly, Mathumathi Rajavel, Kristin M Willner, Amy Butani, Matthew Henry, Biswajit Biswas, Manrong Li, M John Albert, Martin J Loessner, Richard Calendar, Shanmuga Sozhamannan

**Affiliations:** 1 J Craig Venter Institute, Rockville, MD, USA; 2 Institute of Food, Nutrition and Health, ETH Zurich, Schmelzbergstrasse 7, Zurich, 8092, Switzerland; 3 Henry M. Jackson Foundation, Bethesda, MD, USA; 4 Biological Defense Research Directorate, Naval Medical Research Center, Ft. Detrick, MD, USA; 5 Department of Biology/Medical Technology, School of Computer, Mathematical and Natural Sciences, Morgan State University, 1700 E. Cold Spring Lane, Baltimore, MD, USA; 6 Intralytix, Inc., The Columbus Center, 701 E. Pratt St, Baltimore, MD, USA; 7 Department of Microbiology, Faculty of Medicine, Kuwait University, Safat, Kuwait; 8 Department of Molecular and Cell Biology, University of California Berkeley, Berkeley, CA, USA; 9 Current address: GoldBelt Raven, LLC, Frederick, MD, 21701, USA

**Keywords:** *Vibrio cholerae* O139, N4-like virus, Genome comparison, Terminal repeats, Intein, Phylogenetic relationship

## Abstract

**Background:**

*Vibrio cholerae* O139 Bengal is the only serogroup other than O1 implicated in cholera epidemics. We describe the isolation and characterization of an O139 serogroup-specific phage, vB_VchP_VchO139-I (ϕVchO139-I) that has similar host range and virion morphology as phage vB_VchP_JA1 (ϕJA1) described previously. We aimed at a complete molecular characterization of both phages and elucidation of their genetic and structural differences and assessment of their genetic relatedness to the N4-like phage group.

**Methods:**

Host-range analysis and plaque morphology screening were done for both ϕJA1 and ϕVchO139-I. Both phage genomes were sequenced by a 454 and Sanger hybrid approach. Genomes were annotated and protein homologies were determined by Blast and HHPred. Restriction profiles, PFGE patterns and data on the physical genome structure were acquired and phylogenetic analyses were performed.

**Results:**

The host specificity of ϕJA1 has been attributed to the unique capsular O-antigen produced by O139 strains. Plaque morphologies of the two phages were different; ϕVchO139-I produced a larger halo around the plaques than ϕJA1. Restriction profiles of ϕJA1 and ϕVchO139-I genomes were also different. The genomes of ϕJA1 and ϕVchO139-I consisted of linear double-stranded DNA of 71,252 and 70,938 base pairs. The presence of direct terminal repeats of around 1974 base pairs was demonstrated. Whole genome comparison revealed single nucleotide polymorphisms, small insertions/deletions and differences in gene content. Both genomes had 79 predicted protein encoding sequences, of which only 59 were identical between the two closely related phages. They also encoded one tRNA-Arg gene, an intein within the large terminase gene, and four homing endonuclease genes. Whole genome phylogenetic analyses of ϕJA1 and ϕVchO139-I against other sequenced N4-like phages delineate three novel subgroups or clades within this phage family.

**Conclusions:**

The closely related phages feature significant genetic differences, in spite of being morphologically identical. The phage morphology, genetic organization, genomic content and large terminase protein based phylogeny support the placement of these two phages in the *Podoviridae* family, more specifically within the N4-like phage group. The physical genome structure of ϕJA1 could be demonstrated experimentally. Our data pave the way for potential use of ϕJA1 and ϕVchO139-I in *Vibrio cholerae* typing and control.

## Background

Cholera is an acute diarrheal disease caused by ingestion of food or water contaminated with the Gram-negative bacterium *Vibrio cholerae*. These organisms reside and survive within environmental reservoirs during inter-epidemic periods and cause seasonal outbreaks in cholera endemic countries. Cholera is a major concern in parts of the world lacking adequate sanitary infrastructure, especially during mass migration of populations due to human driven or natural disasters, as was evident during the 2010 cholera epidemics in Haiti [1-3]. *V. cholerae* has a short incubation period and produces an enterotoxin that causes a copious, painless, watery diarrhea that can quickly lead to severe dehydration and death, if treatment is not promptly administered. Up to 80% of cases can be successfully treated with oral rehydration salts [4,5]. Since 2000, the incidence of cholera has increased steadily, culminating in 589,854 reported cases from 58 countries worldwide, including 7816 deaths in 2011, with a case-fatality rate (CFR) of 1.3% [4,5]. However, according to the World Health Organization, an estimated 3–5 million cholera cases and 100,000–120,000 deaths occur every year, underscoring the severity of this disease [4,5].

More than 200 serogroups of *V. cholerae* are known [6]. However, all seven pandemics in the recorded history of cholera have been caused by *V. cholerae* strains of the O1 serogroup. The 6^th^ and 7^th^ pandemic O1 strains belong to the Classical and El Tor biotypes, respectively [7]. *V. cholerae* O139 Bengal, a novel pathogen that emerged in late 1992, has remained endemic in South Asia [8]. There is overwhelming genetic evidence supporting the hypothesis that the O139 serogroup emerged from an O1 El Tor strain by horizontal transfer of the O-antigen gene cluster [9,10]. The evolution of *V. cholerae*, as a pathogen, is fairly well understood. Two major virulence factors; CTX (encoding the cholera toxin) and TcpA (encoding the toxin coregulated pilus) and the master regulatory factor, ToxT have been extensively characterized [11,12]. In a landmark study, the genes encoding cholera toxin, *ctx*AB, were shown to be carried on, and horizontally transferred by, a filamentous phage, ϕCTX [13].

Successful epidemiological surveillance and public health intervention of disease outbreaks (such as containment of the disease spread and administration of medical countermeasures) depend on rapid, accurate and inexpensive identification of the etiological agent. Besides the potential therapeutic use of phages, the specificity of phage-bacterial interaction has been exploited for accurate identification of bacteria. Phage-typing schemes have been in use for identification of many bacterial pathogens with high specificity, the most notable being *Salmonella*, *Mycobacterium* and *Listeria*[14-16]. Phage-typing schemes have also been in use for sub typing Classical and El Tor *V. cholerae* strains. These schemes have been periodically updated with new phages to address the changing genetic and epidemiological profiles of *V. cholerae* strains during yearly epidemics [17,18]. During the *V. cholerae* O139 outbreaks of 1992 in the Indian subcontinent, a phage designated as ϕJA1 belonging to the family *Podoviridae*, which specifically infects O139 strains, was isolated in Dhaka [19]. Several additional phages specific for this new pathogen were subsequently isolated, and a new phage typing scheme for *V. cholerae* O139 has been put forth [20].

The *V. cholerae* O139-specific ϕJA1 has been further characterized. It was hypothesized that the O139 O-antigen is the phage receptor since mutants lacking the O-antigen were found to be phage resistant [9,19]. Furthermore, a phage-encoded lyase enzyme was shown to degrade the O139 polysaccharide [21,22]. However, neither has the genome sequence of this phage been determined nor has the gene encoding the lyase enzyme been identified yet.

Here we present the genetic characterization of another *V. cholerae* O139 Bengal-specific phage, ϕVchO139-I, isolated in 1999 and compare it to ϕJA1 isolated in 1992. ϕVchO139-I is a lytic, double-stranded DNA phage, similar but not identical to ϕJA1 [19]. We also present the genomic sequences of phages ϕJA1 and ϕVchO139-I and compare their genetic organization and genomic content to seventeen other currently available phages of the N4-like group.

## Results and discussion

### Isolation and characterization of *V. cholerae* phage ϕVchO139-I

**Figure 1:**
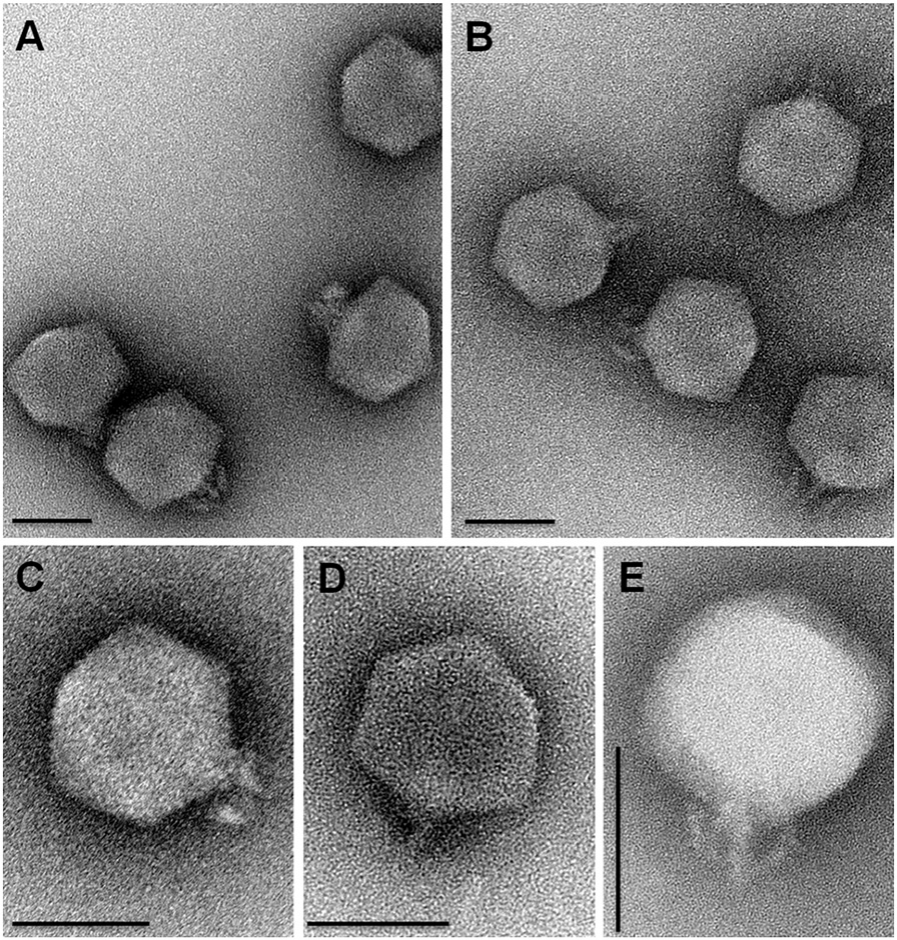
**Transmission electron micrographs of phages ϕJA1 and ϕVchO139-I.** Plates **A**-**D** show images of phage particles negatively stained with 2% uranyl acetate and plate E shows an image of a phage particle negatively stained with 2% phosphotungstate. **A**) ϕJA1. **B**) ϕVchO139-I. **C**) ϕJA1. **D**) ϕVchO139-I. **E**) ϕVchO139-I. Scale bar represents 100 nm. *Podoviridae* morphology of ϕJA1 and ϕVchO139-I particles is evident.

A phage that infects *V. cholerae* O139 Bengal, designated as ϕVchO139-I, was isolated in 1999 from the sewage effluent of the International Centre for Diarrheal Disease Research, Bangladesh (ICDDR,B), hospital in Dhaka. Upon transmission electron microscopic analysis, ϕVchO193-I was found to have similar morphology to a *V. cholerae* phage described earlier, namely ϕJA1 [19]. Both phages featured isometric heads and short non-contractile tails with 6 short fibers, clearly placing them in the *Podoviridae* family of the order *Caudovirales*[23,24]. ϕJA1 and ϕVchO139-I have a capsid diameter of ~68.7 nm and ~64.8 nm, respectively (Figure 1).

ϕJA1 was isolated from the same location seven years prior to the isolation of ϕVchO139-I. Also, ϕVchO139-I has the same host range as ϕJA1. Like ϕJA1, ϕVchO139-I infected all tested strains of *V. cholerae* serogroup O139, but failed to infect any other serogroup strain (serogroup O1-O206) (Additional file 1: Figure S1) or O139 Bengal acapsular mutants (data not shown). ϕJA1 is known to produce a characteristic halo around the plaque postulated to be due to the degradation of O139 capsular polysaccharide by a polysaccharide lyase secreted from phage-infected and lysed bacteria [21,22].

**Figure 2:**
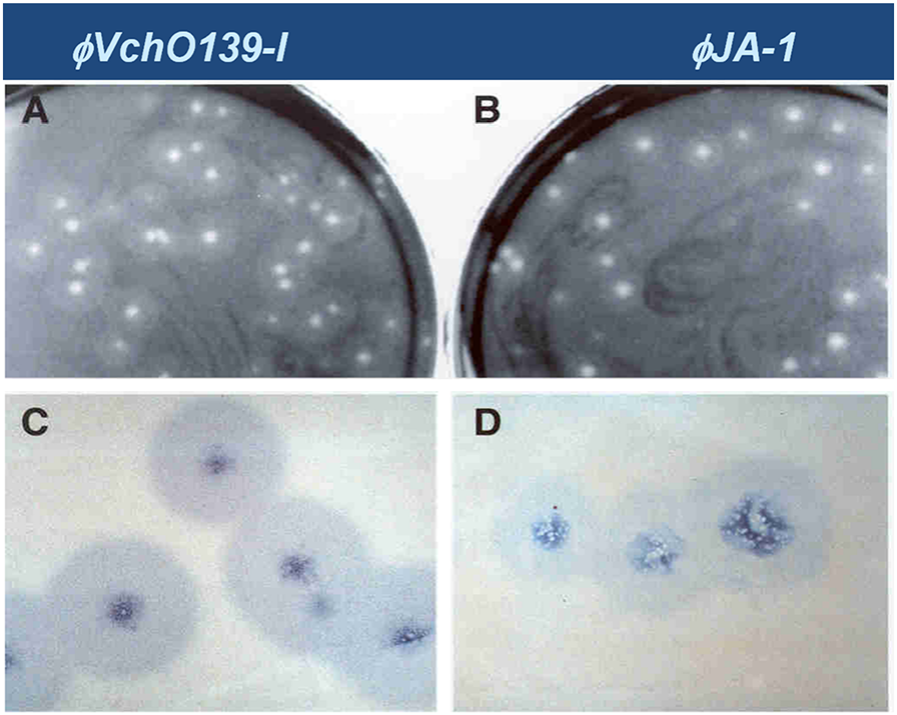
**Plaque morphology of phages ϕJA1 and ϕVchO139-I.** Bacterial lawns of *V. cholerae* O139 Bengal strain AI1834 prepared on 2xYT bottom and top agar show differing plaque morphologies of phages ϕJA1 and ϕVchO139-I. **A**) ϕVchO139-I; **B**) ϕJA1; **C**) ϕVchO139-I close-up view of plaques; **D**) ϕJA1 close-up view of plaques.

Although the virion morphologies of ϕJA1 and ϕVchO139-I were similar, their plaque morphologies were slightly different. Consistently, a larger halo was seen around all the plaques produced by ϕVchO139-I compared with that of ϕJA1 in 2xYT soft agar plates (Figure 2). This difference could be attributed to the genetic differences between the two phages leading to variance in the expression level or the amount or the activity of a secreted lyase. Single-step growth experiments indicated that neither the time of lysis nor the burst size was different for ϕJA1 and ϕVchO139-I. Both phages had a burst size of about 150 and lysis time of about 60 min in LB at 37°C (data not shown).

Based on electron micrographic DNA length measurements, the genome size of ϕVchO139-I was estimated to be 69.9 kb (data not shown). PFGE estimate indicated a genome size of approximately 72 kb for both phages ϕJA1 and ϕVchO139-I (Additional file 1: Figure S2A). Restriction fragment length polymorphism (RFLP) patterns of the genomes of ϕJA1 and ϕVchO139-I with several enzymes were different (*Hpa*II, *ClaI*, *Xmn*I) whereas with some other enzymes (*EcoRI* and *Hind*III) they were identical (Additional file 1: Figure S2B).

### Identification of virion structural proteins

**Figure 3:**
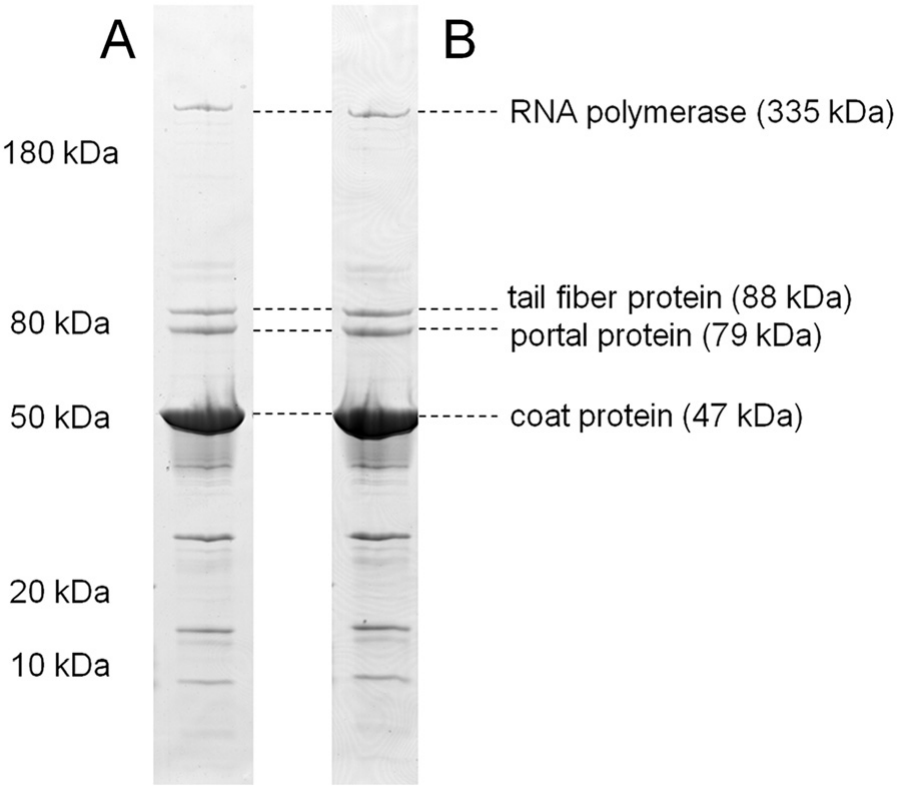
**Identification of virion structural proteins of phages ϕJA1 and ϕVchO139-I.** Proteins were separated on an 8-18% gradient SDS-PAGE (lane A- ϕJA-1 and lane B- ϕVchO139-I) and extracted from gel slices corresponding to specific bands and submitted for LC-ESI-MS/MS peptide mass fingerprinting. Approximate band molecular weight and sequence annotation of corresponding proteins are indicated. Observed molecular mass of the identified proteins is indicated in parenthesis. Other protein bands were contaminated with capsid protein and could not reliably be identified.

The virion protein profiles of the two phages were examined by gradient SDS-PAGE (Figure 3). Both phages exhibited indistinguishable protein profiles. The identity of peptide fragments from SDS-gel bands was elucidated by peptide mass fingerprinting. A corresponding coding sequence could be assigned to four of the eight prominent bands (Figure 3) by using DNA sequence annotation, which was generated subsequently (see below). The largest protein, (encoded by a 9,069 bp gene, JA1_0053 and VCO139_0054 with a predicted mass of about 335 kDa) corresponds to a virion-encapsulated RNA polymerase protein (vRNAP). The presence of this protein is a hallmark of the N4-like phage family [25]. The discrepancy seen between the predicted and observed masses of vRNAP protein could indicate the limitation of size estimation by SDS-PAGE in this size range. The most prominent band was identified as the major capsid protein (encoded by JA1_0059 and VCO139_0060) with a predicted and estimated mass of 47 kDa. The portal protein (encoded by JA_0062 and VCO139_0063) with a predicted and an estimated mass of 79 kDa and a putative phage minor structural protein (encoded by JA_0065 and VCO139_0066) with an estimated and predicted mass of 87 kDa were identified in both phages. The identification of additional bands was obscured by the presence of the heavily overrepresented major capsid protein in bands of lower molecular mass, probably due to trapping effects or degradation processes. Similar difficulties have been observed in other peptide mass fingerprinting experiments in different phages [26].

Despite the fact that phages ϕJA1 and ϕVchO139-I exhibit differences in halo size of their plaques, we did not observe any difference in the relative concentration of any protein in the gel. Based on all these structural characteristics, ϕJA1 and ϕVchO139-I appeared to be very similar phages with minor differences.

### Genome sequencing of ϕJA1 and ϕVchO139I

In order to decipher the genetic differences between the two phages, we carried out whole genome sequencing of the two phages. An initial draft genome of ϕJA1 and ϕVchO139-I was obtained by Sanger-based whole genome shotgun sequencing with finishing reactions performed for ϕJA1. The Sanger assembled single contigs of ϕJA1 and ϕVchO139-I genomes were 69,755 and 68,268 bps in size respectively. We reasoned that the genome was incomplete at this stage because of the discrepancy we observed between the genome sizes of the assembled genomes and the PFGE size estimate (~72 kb) as well as restriction fragments not accounted for by the assembly. Since it is possible that some of the clones were toxic to *E. coli* and hence were excluded, a cloning-independent NextGen sequencing approach was taken to complete the DNA sequencing of these genomes. To accomplish this, an additional 502,102 (ϕJA1) and 507,297 (ϕVchO139-I) 454 pyrosequencing reads were generated. *De novo* assembly of a subset of 15,000 Roche/454 reads each resulted in single contigs of 69,751 bp and 69,354 bp for ϕJA1 and ϕVchO139-I respectively. All 454 and for ϕJA1 additional Sanger reads were mapped back to these contigs. Regions of low coverage were confirmed by Sanger sequencing.

### Determination of the structure and linearity of ϕJA1 and ϕVchO139-I genomic ends

Because the ϕJA1 genome sequence had a better quality assembly, having had more successful finishing reactions and fewer sequence ambiguities, it was used to determine the sequence of the genome ends. The resultant mapping assembly revealed a pile-up of 454 reads spanning the terminal 1,974 base pairs at the 3′ end of the assembly with twice the sequence depth of the remainder of the genome. This suggested that this specific sequence is present twice in the phage genome (Additional file 1: Figure S3), which could explain the size discrepancy. It is well known that N4-like phages have terminal repeats, but they are usually not identical.

Analysis of terminal restriction enzyme fragments and primer walks were used to partially determine the structure of the ϕJA1 genomic ends. We were unable to clone the terminal restriction fragments of the genome in order to obtain sequences corresponding to the putative genomic ends that could be mapped onto the assembly. Bal31 nuclease pre-treatment was used to help identify the true physical ends of the genome as described previously [26]; however, no progressive shortening of any restriction fragment was observed, indicating that the ϕJA1 and ϕVchO139-I genomic ends are inaccessible to Bal31 enzyme. A pre-digestion with proteinase K also did not improve Bal31 accessibility to the ends, indicating the absence of a covalently bound terminal protein (data not shown). It is possible that inter or intra molecular circularization of the genomes prevent Bal31 entry. Hence, at present, the reason for Bal31 resistance of the phage genomic termini is unclear.

**Figure 4:**
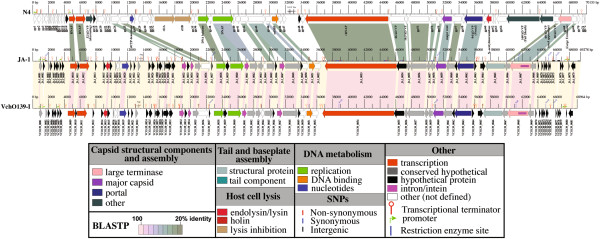
**Genome maps and protein identity comparison between phages N4, ϕJA1, and ϕVchO139-I.** Genetic maps of the genomes of phages N4, ϕJA-1 and ϕVchO139-I. The linear map is based on nucleotide sequences of the phage genomes and predicted open reading frames. CDSs are labeled by locus identifier and colored by functional role categories as noted in the boxed key. BLASTP matches between CDSs are colored by protein percent identity (se e key). Green arrows and red hairpin structures indicate predicted promoters and transcriptional terminators, respectively. Blue vertical lines indicate restriction enzyme recognition sites with the restriction enzyme name marked on top. The location of SNPs is noted on the ϕJA 1 map as small colored tick marks (see key).

Oligonucleotide primers were designed to sequence off the ends of the ϕJA1 genome. The 3′ genomic end was predicted to be contained within a 7.8 kb *Sal*I fragment (see Figure 4 JA-1 map for location of restriction site); therefore, the gel-purified *Sal*I fragment was used as template for sequencing reactions. Primers designed to read off the 3′ end of the genome exhibited a detectable drop in signal intensity, indicating the likely 3′ genome end had been reached (data not shown). The 3′ end terminated with a 12 nucleotide motif (GATAGGGGATAG), an inverted repeat of ′GATAGG′ that is similar to motifs found in other N4-like phages, such as EaS6 [27]. Sequencing off of the predicted 5′ end, using the 5′ 1.6 kb *Nru*I fragment (Figure 4), produced the same 12-nucleotide motif across the junction between the unique sequence and the repeat region at the 5′ end. However, further sequencing using outward primers did not result in any visible loss in signal intensity that would indicate that the terminus had been reached. These results indicated possible contamination of the template with molecules having other topologies such as intra molecular circles or inter molecular dimers of phage DNA. Because of this result, the genome sequence of ϕJA1 is represented with the repeat at only the 3′ end, yielding a total non-redundant genome size of 69,278 bps. The length of the repeat was estimated from the coordinates of the 3′ pile-up to be at least 1,974 bps; therefore, the predicted terminally redundant genome length of ϕJA1 is estimated to be about 71,252 bps. ϕVchO139-I has similar repeats at the ends of the genome and was unresolved at the 5′ terminus, with genome sizes of the non-redundant and terminally redundant genome estimated to be 68,964 bps and about 70,938 bps respectively. These results highlight the inherent difficulty associated with sequencing repeat regions (in this case about 2,000 bps) of more than the average read lengths of the sequencing platforms used in this study. The average Sanger read lengths are about 900 bps and that of 454 around 400–600 bps. It is possible to overcome this problem either by combining paired-end read data or generating read data from platforms such as PacBio that currently produces mean average read lengths of around 4.5 kb [28].

To determine whether the ϕJA1 genome is linear or circularly permuted and whether the ends are fixed or variable, restriction enzyme analyses were performed. Restriction digestion of ϕJA1 DNA produced two distinct fragments when cut with enzymes that have single recognition sites (*Sal*I, *Pau*I and *Nhe*I) and three distinct fragments when cut with enzymes that have two sites such as *Cla*I (data not shown). ϕVchO139-I DNA was cut into two distinct fragments when single cutters *Cla*I and *Pau*I were used (data not shown). Both ϕJA1 and ϕVchO139-I DNAs produced three fragments upon double digestion with enzymes that have a unique restriction site (e.g., *Xho*I-*Ahd*I, *Xho*I-*Sal*I and *Xho*I-*Sph*I) (Additional file 1: Figure S2A). These findings clearly indicate a fixed linear genome structure without circular permutation [26]. Possible cohesive genome ends (*cos*) were examined by heating one half of *Cla*I, *Van*91I and *Oli*I restriction digests for 10 min at 75°C prior to electrophoresis. This heating step did not change the fragment pattern, compared to the unheated control, thus excluding the presence of cohesive genomic ends (data not shown) [29]. This finding is consistent with the placement of these phages in N4-like phage group (see below) in that N4- like phages are not known to have *cos* sites [30].

### ϕJA1 and ϕVchO139-I genome properties and polymorphisms

Despite having been isolated seven years apart, ϕJA1 and ϕVchO139-I have very similar genetic organization and genome sequences. The non-redundant genomes of ϕJA1 and ϕVchO139-I were found to be 69,278 bps and 68,964 bps in length, respectively with an identical G + C mol% of 34.6%, which differs considerably from the G + C mol% of the genome of the host *V. cholerae*, which averages ~47.6% per strain. Direct pairwise comparison of their genomes at the nucleotide level revealed that they are 99% identical across 99% of the length, using NCBI Megablast. The differences were due to a total of 298 single nucleotide polymorphisms (SNPs) and two small insertions/deletions (JA1_0002 and VCO139_0077 have no ortholog in the other genome, Figure 4). The absence of these CDSs in the other genome was confirmed by BLASTN analysis. Of these SNPs, 96 were intergenic, 139 SNPs were synonymous, and 63 resulted in non-synonymous amino acid coding changes in 19 proteins (Figure 4, Additional file 1: Table S1). Additionally, there were 79 predicted protein-coding sequences identified in the genomes of ϕJA1 and ϕVchO139-I, of which 59 are identical between the two phages. Of these, 53 were shown to be 100% identical at the nucleotide level (Additional file 1: Table S1). Both ϕJA1 and ϕVchO139-I encode a single tRNA-Arg gene between JA1_0023 and JA1_0025 in ϕJA1 and VCO139_0022 and VCO139_0024 in ϕVchO139-I and an intein within the large terminase gene.

### Comparison of ϕJA1 and ϕVchO139-I with N4

Top BLASTP matches in the NCBI nr database revealed relatedness to *E. coli* phage N4-like phages. Comparison with *E. coli* phage N4 revealed 22 N4 proteins with BLASTP matches to ϕJA1 and considerable synteny (Figure 4). These matches include functions involved in head morphogenesis (i.e., large terminase, portal, and major coat proteins), transcription (i.e., both N4 RNA polymerase proteins), and replication (i.e., DNA helicase, ssDNA-binding protein (DBP) and DNA polymerase proteins). The presence of the very large (around 335 KDa) virion-encapsulated RNA polymerase (vRNAP) is a hallmark of the N4-like phages as well as the heterodimeric RNA polymerase used in middle mode transcription [31]. Like bacteriophage T4, N4 has homologs of the ‘rapid-lysis’ genes, *rII*A and *rII*B [32,33]; however, both ϕJA1 and ϕVchO139-I lack homologs of these genes. Likewise, homologs of the N4 major tail protein (gp65), neck appendage (gp66), and virion decorating (gp17) proteins appear to be missing from ϕJA1 and ϕVchO139-I as well as an N-acetylmuramidase (gp61). ϕJA1 and ϕVchO139-I also lack tRNAs-Asn, Ser, Thr, and Pro, found between gp47 and gp48 of N4, but instead both encode a single tRNA-Arg gene as noted above. The location of the tRNA genes is different between these *Vibrio* phages and N4.

ϕJA1 and ϕVchO139-I genomes are enriched with selfish DNA compared to N4. Specifically, ϕJA1 and ϕVchO139-I each have an intein within the large terminase protein (JA1_0067) as well as four copies of an HNH family of homing endonuclease (JA1_0032, 0034, 0041, and 0047 of ϕJA1 and VCO139_0032, 0034, 0041 and 0047) (Figure 4 and Additional file 1: Table S1), in contrast to N4, which has none of these selfish DNA elements. Homing endonucleases are site-specific DNA endonucleases that initiate mobility by introducing double-strand breaks at defined positions in genomes lacking the endonuclease gene, stimulating repair and recombination pathways that mobilize the endonuclease coding region [34]. These homing endonucleases typically reside within self-splicing group I introns [35]. Indeed, three of the four HNH endonuclease genes split genes into two parts (N4 homologs gp24, DNAP, and gp43, Figure 4), suggesting that these HNH homing endonucleases may be carried on an intron.

A virion-associated/secreted lyase enzyme encoded by ϕJA1 has been described [21,22]. We identified a candidate gene, JA1_0065 in ϕJA1 (87.3 kDa) and VCO139_0066 in ϕVchO139-I (87.4 kDa), annotated as a putative minor structural protein containing a domain with very weak BLASTP homology to a phage-related tail fiber protein and a Hidden Markov Model match to a peptidase domain (pfam13884) prototypically found in the endosialidase of bacteriophage K1F [36]. The K1F polysialic acid capsule-degrading endosialidase is a key component of the tail spike of phage K1F, and has been observed in other phages [37]. There are six nucleotide differences between the ϕJA1 and ϕVchO139-I minor structural proteins of which five are non-synonymous changes leading to amino acid differences in the proteins. We speculate that this minor structural protein is the polysaccharide lyase enzyme although verification of this prediction awaits further functional genomics experiments.

### N4-like phage family comparisons

To better understand the diversity and relatedness of the N4 family of phages, genomes with top BLAST matches to N4 and falling under the N4-like phage taxonomic classification in the NCBI database were compared. Using N4 as the reference genome, a total of 18 N4-related phages were compared to N4 with an average percent identity (API) at the amino acid level, as high as 65% for *Enterobacter* phage KBNP21 to as low as 43% for *Vibrio* phage ϕJA1 (Additional file 1: Table S2). Of the phages compared, the two *Vibrio parahaemolyticus* phages VBP32 and VBP47 were the closest relatives of ϕJA1 and ϕVchO139-I with an API to N4 of 44% (Additional file 1: Table S2). All these genomes showed considerable synteny, but variable gene conservation (Additional file 1: Figure S4). Every N4-like phage encoded two RNA polymerases, including the vRNAP, making them bona fide members of the N4 family. The presence of *rII*A and *rII*B varied widely across the family, as did the gene content between the portal (JA1_0062) and large terminase (JA1_0067) genes. *E. coli* phage vB_EcoP_G7C had the greatest number of HNH homing endonuclease genes (5), but none of them appear to have disrupted any gene.

**Figure 5:**
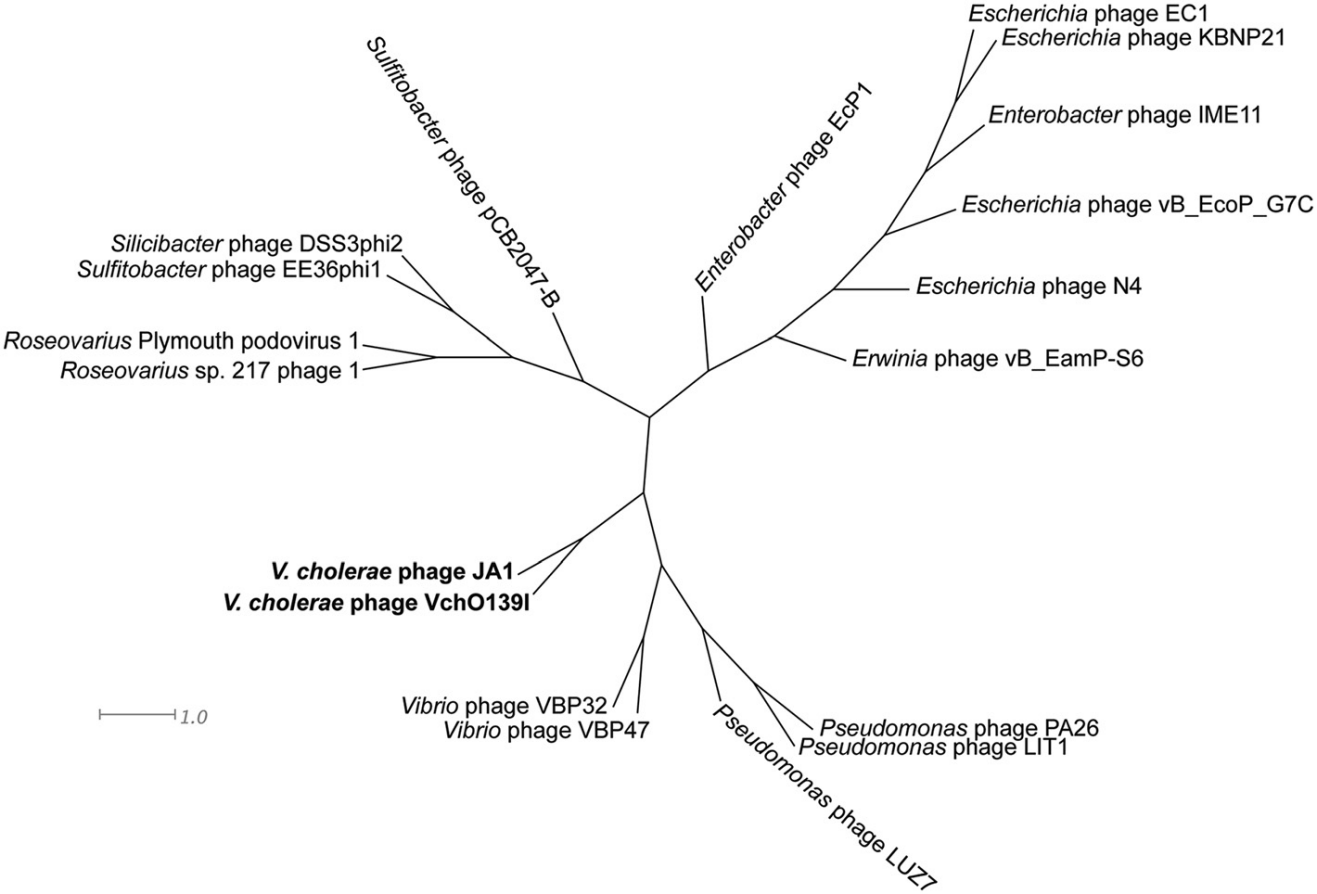
**Proteome-based phylogenetic analysis of N4-like phages.** A dendrogram was constructed based on the mean of the pairwise BLASTP score ratios (BSRs) of the N4-like group of phages.

Since the genomes depicted in Additional file 1: Figure S4 were ordered relative to N4 (from most similar to least similar), and only linear relationships can be observed, a dendrogram (splitstree view) based on the mean of the BLASTP score ratio (BSR) was generated (Figure 5). This tree shows that there were 3 deep branching subgroups or clades of N4-like phages *Vibrio* and *Pseudomonas* phages; enteric phages; and *Roseovarius, Silicibacter* and *Sulfitobacter* phages.

Initial assemblies using 454 sequence reads revealed the presence of apparent frame-shifts caused by variation in homopolymeric tracts that were subsequently corrected by high-quality Sanger data. We wanted to investigate whether this finding (frame-shifts due to homopolymeric tracts) was unique to these *V. cholerae* phages or prevalent in other phages as well. Using the EMBOSS program *fuzznuc*, the number of homopolymeric tracts of greater than 5 nucleotides in length were tabulated for the N4 family of phages and for the *E. coli* tailed phages λ, Mu, P1, T1, T4, T5 and T7 (Additional file 1: Table S3). This data showed that although homopolymeric tracts of up to 8 nucleotides in length are common in these phages, N4-like phages had the longest homopolymeric tracts among the phages searched (up to 12 nucleotides for ϕVchO139-I). The significance of this finding is not clear at this time, although it may be related to the replication mechanism of the N4-like phages or to the regulation of gene expression via a polymerase slippage mechanism.

### ϕJA1 and ϕVchO139-I employ an N4-like DNA packaging mechanism

**Figure 6:**
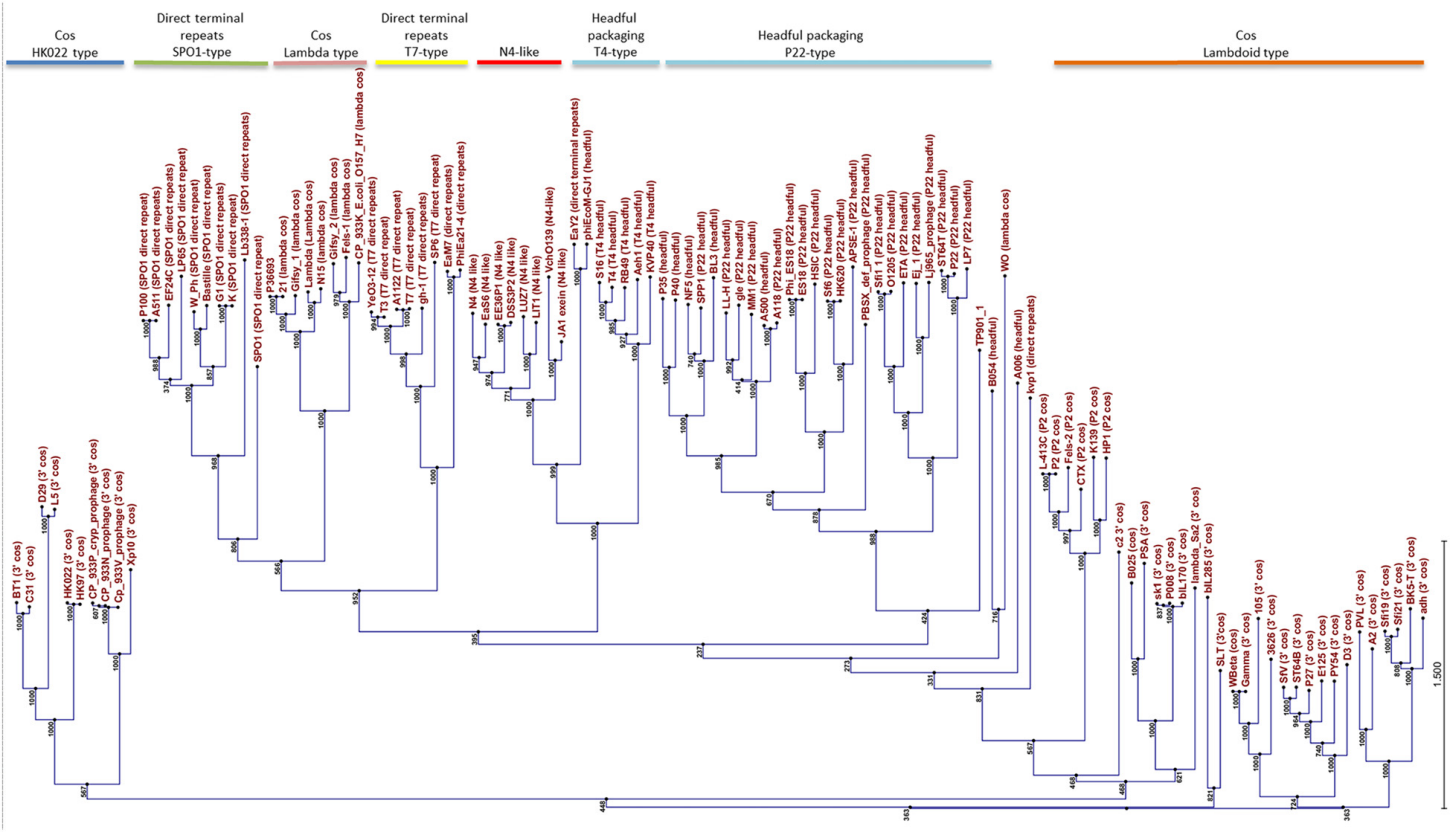
**Phylogenetic tree based on the large terminase protein of various phages.** ClustalW alignment of 109 large terminase subunit amino acid sequences computed into a phylogenetic tree using Neighbor Joining method and 1000 bootstrap replicates (CLC Genomics Workbench 6).

Large terminase protein sequences have been used to construct phylogeny and decipher evolutionary relationships among phages belonging to different families (Casjens et al., 2005). Clustering of the amino acid sequences of the large terminase proteins encoded by ϕJA1 and ϕVchO139-I with that of 109 other phages updated from an earlier study [38], clearly placed both phages within the branch of the N4-like phages (Figure 6). The prototypical N4 phage genome features direct terminal repeats (DTRs), varying in length from 390–440 bps and also contains 3′ single-stranded extensions at the ends of the genome [30]. In other N4-like phages for which the DTRs have been determined, the lengths of the DTRs varied from 397 to 1,120 bps (Additional file 1: Table S3) whereas ϕJA1 and ϕVchO139-I carried by far the longest DTRs of about 1,974 bps.

## Conclusions

Although phages φJA1 and φVchO139-I are 99% identical there are considerable differences between them in terms of single nucleotide polymorphisms and some insertions/deletions. Phages can be considered different when there are discernible phenotypic differences (such as plaque morphologies, growth characteristics, host range etc.) that can be linked to genetic differences. In this case, there is a phenotypic difference in the plaque morphology (different sized halos) of ϕJA1 and ϕVchO139-I and hence they would be considered variants although a genetic link has not been established yet. The genetic characterization and genomic data of ϕJA1 and ϕVchO139-I presented in this study lay the foundation for the development of rapid phage-based diagnostics and therapeutics against this cholera-causing *V. cholerae* strain by providing sequence data and gene targets conferring binding specificity and lysis. The genome annotation indicated the absence of any toxin, virulence or lysogeny associated genes that may preclude the phages’ therapeutic utility. The gene encoding the secreted lyase enzyme [22] responsible for the different sized halos around the plaques could not definitively be identified from nucleotide differences between the two phages or by strong similarity to other known lyases. The lack of extensive similarity further underscores the remarkable genetic diversity seen in the lyase genes in nature [39]. Further work on identifying the lyase gene using a functional genomic approach is underway. From a phylogenetic perspective, comparison of the genomes of all the currently available N4-like phages has further delineated sub clades within this group of the *Podoviridae* family.

## Methods

### Bacterial strains and culture conditions

*V. cholerae* O139 Bengal strain AI1837 or 63–93 (MO45) were used for all phage propagation and large scale preparation of ϕJA1 and ϕVchO139-I lysates. The Shimada serotype collection strains representing serogroups O1 through O206 were used for host range analyses [6]. All strains were grown in LB or BHI; phage assays were done in LB or BHI or 2xYT agar plates with the respective soft agar. All the strains were grown from single colonies in the indicated medium (LB or BHI) in flasks or tubes at 37°C with shaking at ~200 rpm.

### Phage isolation and propagation

Untreated raw sewage for phage isolation was collected from the International Centre for Diarrheal Disease Research, Bangladesh (ICDDR,B) hospital sewer located in Dhaka, and filtered through a Whatman filter to remove large particles. Ninety ml of the filtrate was mixed with 10 ml of 10 × LB and 100 μl of an overnight culture of *V. cholerae* O139 strain was added and grown overnight in a 37°C shaker. The cultures were spun and filtered through a 0.45 μm filter. One hundred μls of ten-fold serial dilutions of the filtrate were mixed with 100 μl of a logarithmic phase culture of *V. cholerae* O139 strain, adsorbed for 20 minutes at 37°C and plated with 4 ml of top agar [40]. The plates were incubated overnight at 37°C.

Single plaques purified from overnight plates were purified twice and then propagated by the confluent lysis method [40,41]. The resulting lysate was further purified by CsCl_2_ gradient centrifugation according to an established protocol [42]. Single step growth experiments were performed as previously described [43,44]. Briefly, host strains were grown to mid-log phase, infected with a multiplicity of infection (MOI) of 10 for 10 min at 37°C in the presence of 1 mM CaCl_2_. Infected cells were centrifuged and washed three times to remove unbound phage. Infected cells were serially diluted and plated using the soft-agar overlay method. Data presented are the results from two independent experiments.

### Phage nomenclature

In accordance with the recent recommendation for bacterial virus nomenclature, we have named the *Vibrio* phages as vB_VchP_JA1 and vB_VchP_VchO139-I [40]. In this scheme, the first part of the designation, vB means ‘virus of bacteria’, the second part, VchP indicates the bacterial host *Vibrio cholerae*, and the family of the phage ‘P’ for *Podoviridae*, and the last part JA1 or VchO139-I indicates the laboratory names. These designations have been used in the title, abstract and GenBank submissions. However, for simplicity, only the laboratory designations ϕJA1 and ϕVchO139-I are used throughout this manuscript.

### Electron microscopy

Purified phage particles were negatively stained with 2% uranyl acetate [45]. Samples were observed in a Tecnai G^2^ Spirit electron microscope at 120 kV equipped with an EAGLE CCD camera (FEI Company, Hillsboro, USA). DNA molecules were partially denatured by heating in formamide and prepared for electron microscopy using a modified cytochrome c spreading technique [46]. Electron micrographs of partially denatured molecules along with a size standard (pBluescript plasmid DNA) were measured and processed as described previously [47].

### Host-range analysis

Host range analysis was done by spot tests [48]. The 206 *V. cholerae* serogroup type strains were grown overnight at 37°C and 10 μl of the overnight cultures were spotted on LB or BHI plates, the spots were allowed to dry, followed by spotting 5 μl containing ~10^8^ PFU total amount of ϕJA1 or ϕVchO139-I phage lysates. Spot tests were also done on bacterial lawns. In some experiments, either overnight cultures or dilutions thereof, were mixed with phages at different MOI and spotted. The plates were incubated at 37°C overnight and the results were scored based on clearing or absence of clearing of bacterial spots. The efficiency of plating (e.o.p) was calculated as the ratio of the titer on a test strain compared to the titer on the strain in which the lysate was prepared. Phages JA1 and VchO139-I were prepared either on AI1837 or 63–93 (MO45). The e.o.p on O139 serogroup strains were 1 whereas in all other serogroup strains the e.o.p was 0.

### Phage DNA extraction and analysis

Bacteriophage DNA was extracted as described previously [26,28,42]. Briefly, phage lysate was treated with DNAse and RNAse and the phage particles were pelleted by high-speed centrifugation. The pellet was resuspended in PBS buffer and phage capsids were lysed by proteinase K and heat treatment. Phage DNA was extracted by organic solvents, as previously described [42].

### Whole genome sequencing of ϕJA1 and ϕVchO139-I

Shotgun sequencing by the Sanger method was done as follows: Five to ten μg of phage DNA was sheared to ~2 kb fragments using a nebulizer (Invitrogen corp). The sheared DNA was end polished and cloned into a pCR Blunt 4 Topo Vector. Plasmid DNA was purified from the clones using the Qiagen Plasmid Miniprep kit (Qiagen, Hilden, Germany) and sequenced using an ABI 3700 sequencer with M13 universal and reverse primers. The resulting sequences were assembled using Sequencher software. Gaps in contigs and low coverage regions were filled by primer walking using intact phage DNA as template.

Whole genome pyrosequencing of phages ϕJA1 and ϕVchO139-I was also done in order to boost the coverage of both phage assemblies and to help fill in unclonable regions that may have been toxic to *E. coli*. The Roche/454 sequencing was done according to the manufacturer’s recommended protocol (FLX Titanium) using a Roche/454 sequencer FLX instrument. Subsets of sequences (15,000) were assembled using the Newbler GS De Novo Assembler version 2.6 [49]. The resulting single assembly of each respective genome was used to map the remaining 454 reads and available Sanger reads using the high throughput ‘Map Reads to Reference’ module in CLC Genomics Workbench version 5.5 (http://www.clcbio.com) using default settings. Sanger reads were used to manually edit the sequence in homopolymeric regions.

### Bioinformatic analyses of sequence data

The final assembled sequence was annotated using the JCVI annotation pipeline as described previously [50]. Protein molecular weights were determined from predicted coding sequences (CDSs) using the EMBOSS program *pepstats*. SNPs between the two phage genomes were identified by mapping the ϕVchO139-I 454-reads onto the non-redundant ϕJA1 genome by first using the high throughput ‘Map Reads to Reference’ module followed by the ‘SNP Detection’ module both in CLC Genomics Workbench version 5.5 (http://www.clcbio.com), using default settings. Homopolymeric tracts were identified by analyzing the underlying sequences for each nucleotide within a tract of six or more A, T, G, or C nucleotides as described previously [51]. SNPs within these homopolymeric tracks were not considered for further analysis. Linear illustrations of multiple phage comparisons were constructed using an in-house (JCVI) PERL script LinearDisplay.pl, showing only pairwise BLASTP results with E-value ≤ 1 × 10^-5^ and percent identity ≥ 35%. Phage phylogenetic trees were constructed using the mean of the BLASTP score ratio as described previously [52]. Comparison of the large terminase protein amino acid sequences was performed as described previously [53] using a dataset modified from the original published set [38]. ClustalW alignments were used to generate a phylogenetic tree with the Neighbor Joining method and 1000 bootstrap replicates.

### SDS-PAGE and peptide mass fingerprinting

Peptide mass fingerprinting was performed as described previously [54,55]. Briefly, purified phage particles were collected by centrifugation and mixed with an equal volume of sample buffer prior to heating at 95°C for 5 min. Proteins were separated on precast, horizontal SDS-PAGE gels (Excel Gel gradient 8-18% PAGE gels, GE Healthcare, Solingen, Germany), followed by staining with Coomassie blue R-350 (Phastblue R; GE Healthcare). Major bands were excised from the gel; the gel pieces were washed twice in 100 mM NH_4_HCO_3_/50% acetonitrile and washed once with 50 ul acetonitrile. Supernatants were discarded and proteins in gel fragments were digested with trypsin at 37°C overnight. Supernatants were removed and the gel pieces were extracted twice in 0.1% TFA/50% acetonitrile. Supernatants were dried and dissolved in 0.1% formic acid and analyzed by LC-ESI-MS/MS (Functional Genomics Center, Zurich, Switzerland) and compared against a database of all predicted phage proteins using Scaffold software version 3 (Proteome Software Inc., Portland, OR, USA). Protein domains were predicted with InterProScan (http://www.ebi.ac.uk/InterProScan).

### Determination of the structure of genomic ends

The physical genome structure (linear vs circular) of ϕJA1 and ϕVchO139-I was assessed by restriction digestion profiles under conditions recommended by the manufacturers (New England Biolabs, Ipswich, MA and Fermentas-Thermo Scientific, Waltham, MA). Bal31-nuclease digestion was performed as previously described [26]. The presence of putative cohesive genomic ends was evaluated by digesting genomic DNA with *Cla*I, *Van*91I and *Oli*I, heating half of the digest to 75°C for 10 min and comparing it to the non-heated restriction digests after electrophoresis in an agarose gel. Pulsed-field gel electrophoresis was done using a CHEF-DR-III apparatus (BioRad, Hercules, CA) under the following conditions: Gels (1%) were cast using Megabase agarose (BioRad) and run at 14°C buffer temperature with 1-25 s switch time, 6 V/cm and 120° angle for 17.5 h. PFGE MidRange Marker I and II (New England Biolabs, Ipswich, MA) were used for estimating fragment sizes. Primers for genome-end runoff sequencing were designed based on the consensus genome sequence and Sanger sequencing reactions were carried out using either intact ϕJA1 DNA or isolated restriction fragments as templates. The resulting sequence reads were manually verified by Sequiserve GmbH, Vaterstetten, Germany. Sequencing reactions were performed at the highest possible annealing temperatures in order to prevent potential re-circularization of the template DNA molecule. Phage ϕJA1 genome-end fragments resulting from restriction digestion with *Sal*I or *Nru*I restriction enzyme were cut out of agarose gels and extracted using QiaQuick Gel Extraction Kit (Qiagen, Hilden, Germany). Isolated fragments were checked for purity by electrophoresis and used as templates for Sanger sequencing.

### Nucleotide sequence accession numbers

Complete nucleotide sequences of ϕJA1 and ϕVchO139-I were deposited to GenBank under accession numbers KC438282 and KC438283 respectively. The following seventeen N4-like phage genomes were obtained from NCBI for use in whole genome comparisons: 1) *E. coli* phage KBNP21, JX415535 [56]; 2) *E. coli* phage N4, EF056009 (unpublished); 3) *E. coli* phage vB_EcoP_G7, HQ259105 [57]; 4) *Enterobacter* phage EcP1, HQ641380 (unpublished); 5) *E. coli* phage IME11, JX880034 [58]; 6) *Erwinia* phage vB_EamP-S6, HQ728266 [33] 7) *Pseudomonas* phage LIT1, FN422399; 8) *Pseudomonas* phage LUZ7, FN422398 [59]; 9) *Roseovarius* sp. 217 phage 1, FR682616 (unpublished); 10) *Silicibacter* phage DSS3phi2, FJ591093 and 11) *Sulfitobacter* phage EE36phi1, FJ591094 [60]; 12) *Roseovarius* Plymouth Podovirus 1, FR719956; 13) *Pseudomonas* phage PA26, JX194238 [59]; 14) *Vibrio* phage VBP32, HQ634196 (unpublished); 15) *Vibrio* phage VBP47, HQ634194 (unpublished); 16) *Sulfitobacter* phage pCB2047-B, HQ317387 (unpublished) and 17) *E. coli* phage EC1, KC206276 (unpublished).

## Competing interests

The authors declare that they have no competing interests.

## Authors’ contributions

SS, and MJA conceived the project; SS, MJA, MJL, DEF, JK and RC designed experiments; SS, RC, MR, JK, MH, and BB performed experiments; SS and ML did Shotgun cloning and Sanger sequencing; KABL, KMW and AB did 454 sequencing; DEF, KABL, JK and SS did post sequence analysis; SS, JK, DEF wrote the manuscript and KABL and MJA edited the manuscript. All authors read and approved the final manuscript.

## Supplementary Material

Additional file 1: Figure S1 Host range analysis of phage VchO139-1 by spot test. Ninety-six bacterial cultures of different *V. cholerae* strains were mixed with ϕVchO139-I and spotted using a sterile 96 pronged stainless steel replicator. Plate on the left shows the spots of cultures with buffer and on the right shows the spots of cultures mixed with phage. Complete lysis of the *V. cholerae* strain O139 spot by phage VchO139-I is indicated by an underline in the image whereas all other spots show normal growth in the presence of phage. **Figure S2A.** Determination of the size, linearity and variability of the genomes of phages ϕJA1 and ϕVchO139-I. Pulse field gel electrophoresis of uncut genomic DNAs of phages ϕJA1 (c) and ϕVchO139-I (d). a and b: NEB Mid-Range PFG markers I and II. Approximate band sizes are indicated. In e) the number of fragments produced by double digestion of ϕJA1 DNA with two restriction enzymes are shown; lane 1: Uncut JA1; lane 2: JA1 (*Xho-PstI*); lane 3: JA1 (*Xho*I-*Sal*I). **Figure S2B.** Restriction fragment length polymorphism analysis of ϕJA1 and ϕVchO139-I DNAs. Genomic DNAs of phages ϕJA1 and ϕVchO139-I were digested with the indicated enzymes and run on an agarose gel. The restriction patterns generated by *Eco*RI and *Hind*III are identical whereas that of *Hpa*II and *Xmn*I show some differences. **Figure S3.** Pile-up of sequence reads at the 3′ end of the non-redundant genome assembly. Sanger sequencing based ϕJA1 assembly was used as the reference for mapping the 454 sequence reads. The depth of coverage (approx. 3500-fold) of around 1974 bases at the 3′ end is represented two fold more than the rest of the genome (1800-fold) as is evident by the sharp increase in coverage at this location marked by the arrow. **Figure S4.** Genome maps and protein identity comparison between N4-like phages. Physical map of the genomes of the N4-like phages. The linear map is based on nucleotide sequences of the phage genomes and predicted open reading frames. CDSs are labeled by locus identifier and colored by functional role categories as noted in the boxed key. BLASTP matches between CDSs are colored by protein percent identity (see key). Genomes are ordered from most to least similar to bacteriophage N4. **Table S1.** Comparison of bacteriophages ϕJA1 and ϕVchO139-l. **Table S2.** Average Percent Identity of N4-like Phages at the Amino Acid Level. **Table S3.** Comparison of Miscellaneous features between N4-like Phages and Coliphages. Click here for file
