# Supplementary material for: Whole genome sequencing and comparative genomic analyses of two Vibrio cholerae O139 Bengal-specific Podoviruses to other N4-like phages reveal extensive genetic diversity
Source: Virol J. 2013 May 28;10:165. doi: 10.1186/1743-422X-10-165 (PMC3670811; doi:10.1186/1743-422X-10-165)
Supplement: Additional file 1: Figure S1 — Host range analysis of phage VchO139-1 by spot test. Ninety-six bacterial cultures of different V. cholerae strains were mixed with ϕVchO139-I and spotted using a sterile 96 pronged stainless steel replicator. Plate on the left shows the spots of cultures with buffer and on the right shows the spots of cultures mixed with phage. Complete lysis of the V. cholerae strain O139 spot by phage VchO139-I is indicated by an underline in the image whereas all other spots show normal growth in the presence of phage. Figure S2A. Determination of the size, linearity and variability of the genomes of phages ϕJA1 and ϕVchO139-I. Pulse field gel electrophoresis of uncut genomic DNAs of phages ϕJA1 (c) and ϕVchO139-I (d). a and b: NEB Mid-Range PFG markers I and II. Approximate band sizes are indicated. In e) the number of fragments produced by double digestion of ϕJA1 DNA with two restriction enzymes are shown; lane 1: Uncut JA1; lane 2: JA1 (Xho-PstI); lane 3: JA1 (XhoI-SalI). Figure S2B. Restriction fragment length polymorphism analysis of ϕJA1 and ϕVchO139-I DNAs. Genomic DNAs of phages ϕJA1 and ϕVchO139-I were digested with the indicated enzymes and run on an agarose gel. The restriction patterns generated by EcoRI and HindIII are identical whereas that of HpaII and XmnI show some differences. Figure S3. Pile-up of sequence reads at the 3′ end of the non-redundant genome assembly. Sanger sequencing based ϕJA1 assembly was used as the reference for mapping the 454 sequence reads. The depth of coverage (approx. 3500-fold) of around 1974 bases at the 3′ end is represented two fold more than the rest of the genome (1800-fold) as is evident by the sharp increase in coverage at this location marked by the arrow. Figure S4. Genome maps and protein identity comparison between N4-like phages. Physical map of the genomes of the N4-like phages. The linear map is based on nucleotide sequences of the phage genomes and predicted open reading frames. CDSs are labeled by locus identifier and [file 1743-422X-10-165-S1.pdf]

Figure S1

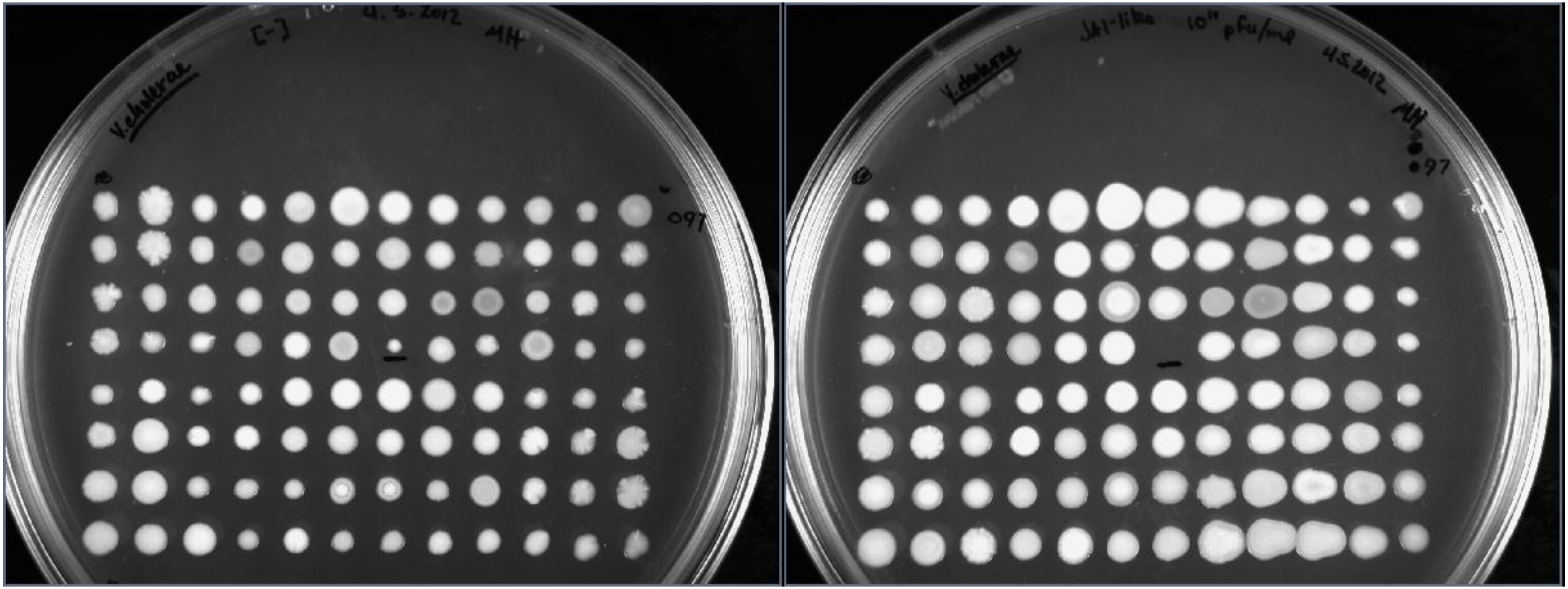

Figure S2

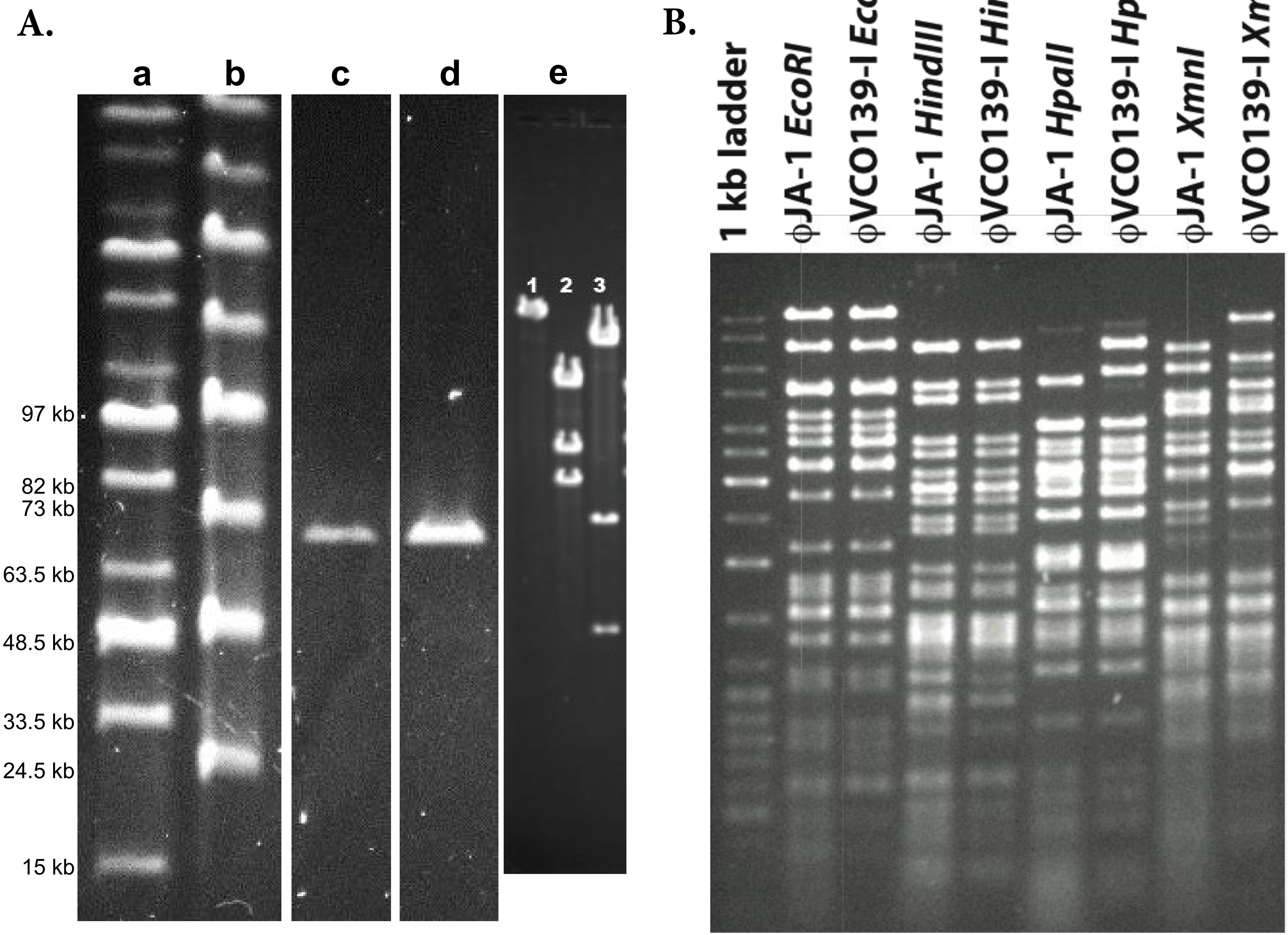
A.
a
b
c
d
e
97 kb
82 kb
73 kb
63.5 kb
48.5 kb
33.5 kb
24.5 kb
15 kb
1
2
3
B.
1 kb ladder
φJA-1 EcoRI
φVCO139-I EcoRI
φJA-1 HindIII
φVCO139-I HindIII
φJA-1 HpaII
φVCO139-I HpaII
φJA-1 XmnI
φVCO139-I XmnI

Figure S3

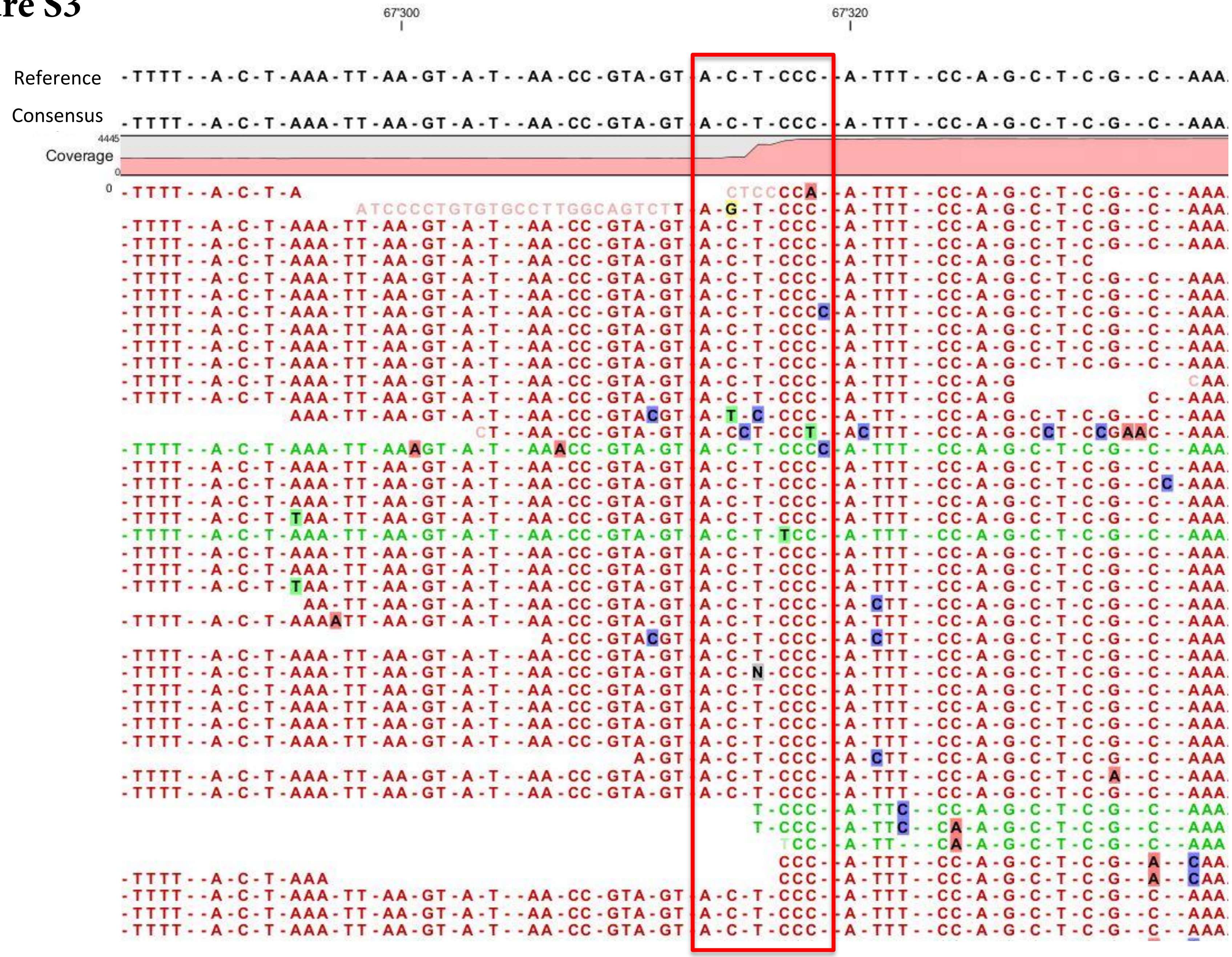

Figure S4

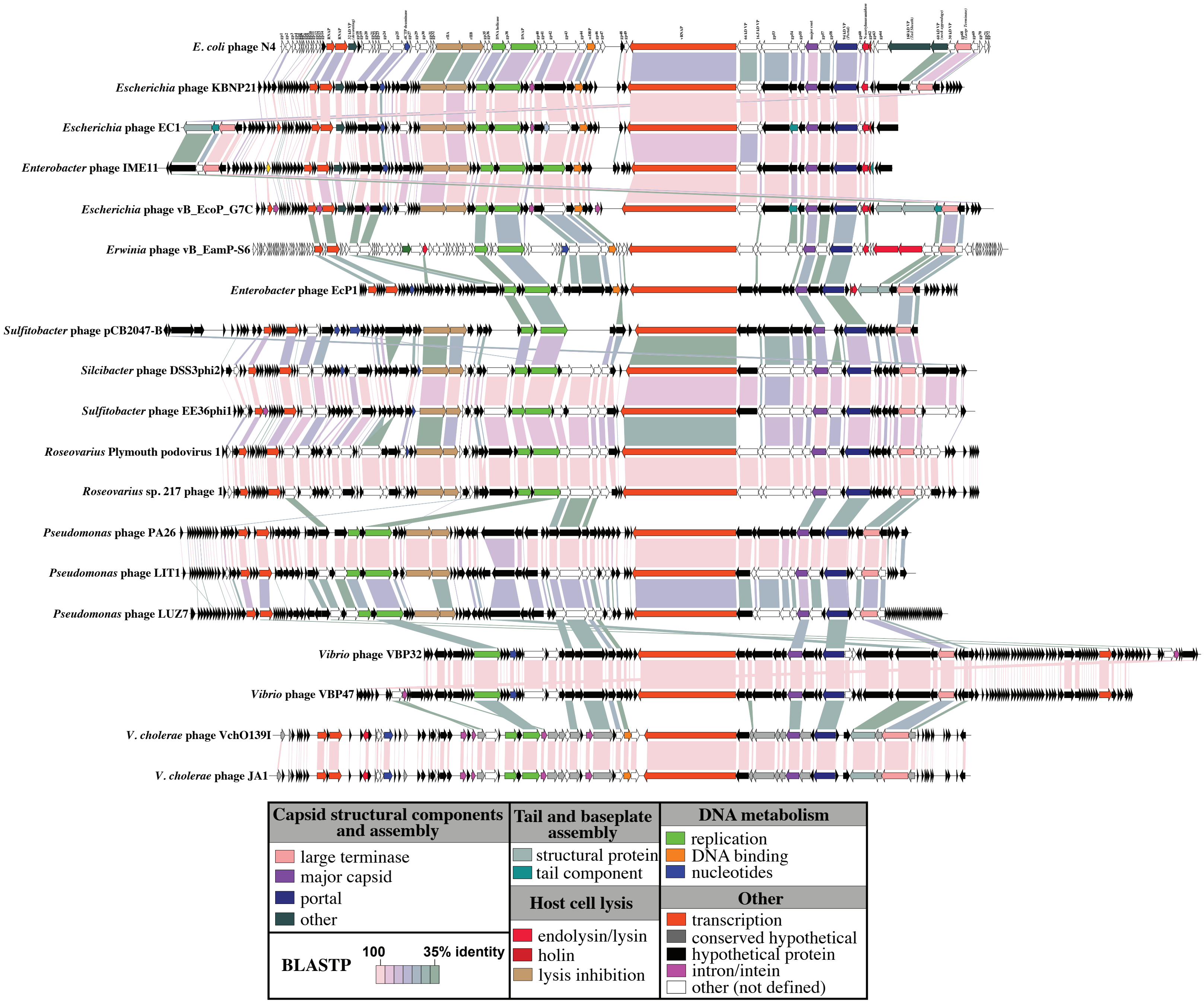

E. coli phage N4
Escherichia phage KBNP21
Escherichia phage EC1
Enterobacter phage IME11
Escherichia phage vB_EcoP_G7C
Erwinia phage vB_EamP-S6
Enterobacter phage EcP1
Sulfitobacter phage pCB2047-B
Silcibacter phage DSS3phi2
Sulfitobacter phage EE36phi1
Roseovarius Plymouth podovirus 1
Roseovarius sp. 217 phage 1
Pseudomonas phage PA26
Pseudomonas phage LIT1
Pseudomonas phage LUZ7
Vibrio phage VBP32
Vibrio phage VBP47
V. cholerae phage VchO139I
V. cholerae phage JA1
Capsid structural components and assembly
large terminase
major capsid
portal
other
Tail and baseplate assembly
structural protein
tail component
Host cell lysis
endolysin/lysin
holin
lysis inhibition
DNA metabolism
replication
DNA binding
nucleotides
Other
transcription
conserved hypothetical
hypothetical protein
intron/intein
other (not defined)
BLASTP
100
35% identity

Table S1. Comparison of Bacteriophages vB_VchP_JA1 and vB_VchP_VchO139-I

vB_VchP_JA1			Mol. Wt.	vB_VchP_VchO139-I			Mol Wt.	BLASTP	BLASTN	SNPs		
Locus	Annotation	Size (bp)	(kDa)	Locus	Annotation	Size (bp)	(kDa)	% id	%id	S	NS	Total (S + NS)
JA1_0001	conserved hypothetical protein	411	15.2	VCO139_0001	conserved hypothetical protein	411	15.2	100	100			
JA1_0002	hypothetical protein	228	9.1									
JA1_0003	conserved hypothetical protein	279	10.6	VCO139_0002	conserved hypothetical protein	279	10.6	100	100			
JA1_0004	hypothetical protein	237	9.1	VCO139_0003	hypothetical protein	237	9.1	100	100			
JA1_0005	hypothetical protein	258	10.0	VCO139_0004	hypothetical protein	258	10.0	100	100			
JA1_0006	hypothetical protein	189	7.4	VCO139_0005	hypothetical protein	189	7.4	100	100			
JA1_0007	hypothetical protein	189	7.3	VCO139_0006	hypothetical protein	189	7.3	100	100			
JA1_0008	hypothetical protein	234	8.6	VCO139_0007	hypothetical protein	234	8.6	100	100			
JA1_0009	hypothetical protein	267	9.6	VCO139_0008	hypothetical protein	267	9.6	100	100			
JA1_0010	DNA-directed RNA polymerase RNAP1	942	36.4	VCO139_0009	DNA-directed RNA polymerase RNAP1	942	36.4	100	100			
JA1_0011	hypothetical protein	216	8.7	VCO139_0010	hypothetical protein	216	8.7	100	100			
JA1_0012	DNA-directed RNA polymerase RNAP2	1227	46.4	VCO139_0011	DNA-directed RNA polymerase RNAP2	1227	46.5	99.3	98.5	15	4	19
JA1_0013	hypothetical protein	189	7.5	VCO139_0012	hypothetical protein	189	7.5	100	100			
JA1_0014	N-acetylmuramoyl-L-alanine amidase	525	19.4	VCO139_0013	N-acetylmuramoyl-L-alanine amidase	525	19.4	100	99.8	1	0	1
JA1_0015	hypothetical protein	273	10.3	VCO139_0014	hypothetical protein	273	10.3	100	100			
JA1_0016	hypothetical protein	297	11.2	VCO139_0015	hypothetical protein	297	11.2	96.9	96.3	6	5	11
JA1_0017	conserved hypothetical protein	129	5.0	VCO139_0016	conserved hypothetical protein	129	5.0	100	100			
JA1_0018	PF10947 family protein	420	16.2	VCO139_0017	PF10947 family protein	420	16.2	100	98.3	5	0	5
JA1_0019	putative lipoprotein	303	11.7	VCO139_0018	putative lipoprotein	303	11.7	100	100			
JA1_0020	thymidylate synthase	852	32.6	VCO139_0019	thymidylate synthase	852	32.6	100	100			
JA1_0021	hypothetical protein	159	6.2	VCO139_0020	hypothetical protein	159	6.2	100	100			
JA1_0022	hypothetical protein	216	7.9	VCO139_0021	hypothetical protein	216	7.9	100	100			
JA1_0023	conserved hypothetical protein	327	12.6	VCO139_0022	conserved hypothetical protein	327	12.7	99.1	99.7	0	1	1
JA1_0025	hypothetical protein	312	11.3	VCO139_0024	hypothetical protein	312	11.3	100	100			
JA1_0026	hypothetical protein	399	15.4	VCO139_0025	hypothetical protein	399	15.4	100	100			
JA1_0080	hypothetical protein	141	5.7	VCO139_0026	hypothetical protein	141	5.7	100	100			
JA1_0027	hypothetical protein	456	18.1	VCO139_0027	hypothetical protein	456	18.1	100	100			
JA1_0028	PF11753 family protein	294	11.5	VCO139_0028	PF11753 family protein	294	11.5	100	100			
JA1_0029	hypothetical protein	183	7.1	VCO139_0029	hypothetical protein	183	7.1	100	100			
JA1_0030	hypothetical protein	642	23.5	VCO139_0030	hypothetical protein	642	23.4	99.5	99.8	0	1	1
JA1_0031	hypothetical protein	456	17.7	VCO139_0031	hypothetical protein	456	17.7	100	99.8	1	0	1
JA1_0032	putative HNH homing endonuclease	576	22.3	VCO139_0032	putative HNH homing endonuclease	576	22.3	100	99.8	1	0	1
JA1_0033	hypothetical protein	321	11.7	VCO139_0033	hypothetical protein	321	11.7	100	100			
JA1_0034	putative HNH homing endonuclease	387	14.4	VCO139_0034	putative HNH homing endonuclease	387	14.4	100	100			
JA1_0035	conserved hypothetical protein	732	27.1	VCO139_0035	conserved hypothetical protein	732	27.1	100	100			
JA1_0036	metallopeptidase domain protein	1230	46.7	VCO139_0036	metallopeptidase domain protein	1230	46.7	100	100			
JA1_0037	hypothetical protein	168	7.1	VCO139_0037	hypothetical protein	168	7.1	100	100			
JA1_0038	DNA helicase	1191	45.3	VCO139_0038	DNA helicase	1191	45.3	100	100			
JA1_0039	hypothetical protein	450	17.6	VCO139_0039	hypothetical protein	450	17.6	99.3	99.6	1	1	2
JA1_0040	DNA polymerase	1680	63.8	VCO139_0040	DNA polymerase	1680	63.8	99.8	99.6	6	1	7
JA1_0041	putative HNH homing endonuclease	534	20.4	VCO139_0041	putative HNH homing endonuclease	534	20.4	100	100			
JA1_0042	conserved hypothetical protein	999	38.1	VCO139_0042	conserved hypothetical protein	999	38.1	100	100			
JA1_0043	conserved hypothetical protein	609	23.4	VCO139_0043	conserved hypothetical protein	609	23.4	100	100			
JA1_0044	phosphoribosyl-ATP diphosphatase	450	16.9	VCO139_0044	phosphoribosyl-ATP diphosphatase	450	16.9	100	100			
JA1_0045	conserved hypothetical protein	1014	38.1	VCO139_0045	conserved hypothetical protein	1014	38.1	100	100			
JA1_0046	hypothetical protein	243	8.9	VCO139_0046	hypothetical protein	243	8.9	100	100			
JA1_0047	putative HNH homing endonuclease	636	23.9	VCO139_0047	putative HNH homing endonuclease	636	23.9	100	100			
JA1_0048	conserved hypothetical protein	1923	73.5	VCO139_0048	conserved hypothetical protein	1923	73.5	100	100			
JA1_0049	hypothetical protein	288	10.6	VCO139_0049	hypothetical protein	288	10.6	100	99.7	1	0	1
JA1_0050	AAA domain protein	738	28.0	VCO139_0050	AAA domain protein	738	28.0	100	100			
JA1_0051	ssDNA binding protein	723	26.1	VCO139_0051	ssDNA binding protein	723	26.1	100	100			
JA1_0052	crossover junction endodeoxyribonuclease RusA	798	30.5	VCO139_0052	crossover junction endodeoxyribonuclease RusA	798	30.5	98.1	99.1	2	5	7
JA1_0053	viron-encapsulated RNA polymerase	9069	335.3	VCO139_0054	viron-encapsulated RNA polymerase	9069	335.4	99.3	99.1	58	21	79
JA1_0054	hypothetical protein	1269	47.2	VCO139_0055	hypothetical protein	1269	47.2	99.3	98.8	12	3	15
JA1_0055	conserved domain protein	408	15.3	VCO139_0056	conserved domain protein	408	15.3	100	100			
JA1_0056	conserved domain protein	2109	79.6	VCO139_0057	conserved domain protein	2109	79.6	99.9	99.7	6	1	7
JA1_0057	conserved hypothetical protein	477	17.1	VCO139_0058	conserved hypothetical protein	477	17.1	100	100			
JA1_0058	conserved hypothetical protein	585	22.2	VCO139_0059	conserved hypothetical protein	585	22.2	100	98.6	8	0	8
JA1_0059	major capsid protein, N4-gp56 family	1314	47.0	VCO139_0060	major capsid protein, N4-gp56 family	1314	47.0	100	100			
JA1_0060	conserved hypothetical protein	1092	40.4	VCO139_0061	conserved hypothetical protein	1092	40.4	99.4	99.5	3	2	5
JA1_0061	hypothetical protein	312	11.7	VCO139_0062	hypothetical protein	312	11.7	98.1	98.1	4	2	6
JA1_0062	portal protein	2118	79.3	VCO139_0063	portal protein	2118	79.3	99.6	99.6	5	3	8
JA1_0063	hypothetical protein	288	11.0	VCO139_0064	hypothetical protein	288	10.9	96.8	99	0	3	3
JA1_0064	hypothetical protein	624	23.4	VCO139_0065	hypothetical protein	624	23.4	99.5	99.8	0	1	1
JA1_0065	putative phage minor structural protein	2427	87.3	VCO139_0066	putative phage minor structural protein	2427	87.4	99.5	99.8	1	5	6
JA1_0066	conserved domain protein	687	25.8	VCO139_0067	conserved hypothetical protein	687	25.9	99.1	99.3	3	2	5
JA1_0067	terminase, large subunit (intein-containing)	2613	99.5	VCO139_0068	terminase, large subunit (intein-containing)	2613	99.5	100	100			
JA1_0068	conserved hypothetical protein	678	25.2	VCO139_0069	conserved hypothetical protein	678	25.2	100	100			
JA1_0069	hypothetical protein	138	5.4	VCO139_0070	hypothetical protein	138	5.4	97.8	99.3	0	1	1
JA1_0070	hypothetical protein	183	7.3	VCO139_0071	hypothetical protein	183	7.3	100	100			
JA1_0071	hypothetical protein	114	4.6	VCO139_0072	hypothetical protein	114	4.6	100	100			
JA1_0072	hypothetical protein	174	7.2	VCO139_0073	hypothetical protein	174	7.2	100	100			
JA1_0073	hypothetical protein	207	8.2	VCO139_0074	hypothetical protein	207	8.2	98.5	99.5	0	1	1
JA1_0074	hypothetical protein	192	7.5	VCO139_0075	hypothetical protein	192	7.5	100	100			
JA1_0075	hypothetical protein	126	5.0	VCO139_0076	hypothetical protein	126	5.0	100	100			
				VCO139_0077	hypothetical protein	132	5.3					
JA1_0076	hypothetical protein	240	9.2	VCO139_0078	hypothetical protein	240	9.2	100	100			
JA1_0077	hypothetical protein	222	8.6	VCO139_0079	hypothetical protein	222	8.6	100	100			
JA1_0078	hypothetical protein	114	4.7	VCO139_0080	hypothetical protein	114	4.7	100	100			
JA1_0079	hypothetical protein	534	20.3	VCO139_0081	hypothetical protein	534	20.3	100	100			
	**Average:**	**699**			**Average:**	**698**			**Totals:**	**139**	**63**	**202**

**# proteins**	**19**	**19**
**Synonymous SNPs**	**139**	
**Non-synonymous SNPs**	**63**	
**intergenic SNPs**	**96**	
**total SNPs**	**298**	

Table S2. Average Percent Identity of N4-like Phages at the Amino Acid Level

	Escherichia phage N4	*Escherichia* phage KBNP21	*Escherichia* phage EC1	*Enterobacter* phage IME11	*Escherichia* phage vB_EcoP_G7C	*Sulfitobacter* phage pCB2047-B	*Pseudomonas* phage LIT1	*Erwinia* phage vB_EamP-S6	*Sulfitobacter* phage EE36phi1	*Pseudomonas* phage PA26	*Silicibacter* phage DSS3phi2	*Enterobacter* phage EcP1	*Pseudomonas* phage LUZ7	*Roseovarius* Plymouth podovirus 1	*Roseovarius* sp. 217 phage 1	*Vibrio* phage VBP32	*Vibrio* phage VBP47	*V. cholerae* phage JA1	*V. cholerae* phage VchO139I
***Escherichia* phage N4**	**100.0**	65.2	64.4	64.2	63.2	48.0	47.7	47.0	47.0	46.5	46.1	45.7	45.4	45.1	45.1	44.0	44.0	42.5	42.5
***Enterobacter* phage IME11**		**100.0**	92.1	88.8	87.0	45.6	44.7	46.2	44.0	44.8	44.9	45.4	46.6	44.2	44.3	44.4	44.4	42.3	42.2
***Escherichia* phage vB_EcoP_G7C**			**100.0**	87.5	85.7	45.6	44.7	46.8	44.0	44.7	44.2	45.2	46.4	44.3	44.3	43.5	43.5	42.3	42.3
***Escherichia* phage KBNP21**				**100.0**	84.4	45.7	44.9	46.4	43.8	45.0	44.6	45.4	46.3	43.8	43.8	44.3	44.3	41.3	41.2
***Escherichia* phage EC1**					**100.0**	45.5	44.9	45.9	44.0	44.9	44.7	45.0	46.4	43.6	43.6	44.1	44.1	42.0	42.0
***Erwinia* phage vB_EamP-S6**						**100.0**	46.6	46.6	59.2	46.4	58.8	45.1	44.9	56.6	56.5	43.6	43.6	39.1	39.2
***Silicibacter* phage DSS3phi2**							**100.0**	45.7	46.1	92.8	45.3	44.4	56.5	45.5	45.5	45.4	45.4	45.1	45.1
***Enterobacter* phage EcP1**								**100.0**	46.2	46.1	46.4	46.1	47.3	44.9	46.0	45.4	45.4	43.7	43.6
***Roseovarius* Plymouth podovirus 1**									**100.0**	44.1	90.1	42.3	43.1	73.1	72.4	43.9	43.9	40.4	40.5
***Roseovarius* sp. 217 phage 1**										**100.0**	43.7	44.4	58.4	44.1	44.1	45.3	45.3	44.9	45.0
***Sulfitobacter* phage EE36phi1**											**100.0**	42.4	42.9	72.7	72.0	44.3	44.3	40.2	40.2
***Pseudomonas* phage LIT1**												**100.0**	45.4	43.3	42.3	43.4	43.4	41.9	41.9
***Pseudomonas* phage PA26**													**100.0**	42.8	42.9	47.1	47.1	45.6	45.6
***Pseudomonas* phage LUZ7**														**100.0**	99.3	44.2	44.2	39.9	40.0
***Vibrio* phage VBP32**															**100.0**	44.2	44.2	39.9	40.0
***Vibrio* phage VBP47**																**100.0**	99.6	44.6	44.5
***Sulfitobacter* phage pCB2047-B**																	**100.0**	44.6	44.5
***V. cholerae* phage JA1**																		**100.0**	99.7
***V. cholerae* phage VchO139I**																			**100.0**

Table S3. Comparison of Miscellaneous features between N4-like Phages and Coliphages

Phage	Accession	Size (bp)	Length of DTRs (bp)	# tRNAs	tRNA type*	#homing endo	#>5 polyA	#>5 polyT	#>5 polyG	#>5 polyC	Total	#HT/bp	pA/#	pT/#	pG/#	pC/#
E. coli phage KBNP21	JX415535	69855	Not Determined	1	Ile	1	11	11	2	1	25	0.000357884	7/1	7/3	6/2	7/1
E. coli phage N4	EF056009	70153	390-440 + 3' ss extension	4	Asn,Ser,Thr,Pro	1	11	13	0	1	25	0.000356364	8/1	7/2	0	9/1
E. coli phage EC1	KC206276	70831	Not Determined	0	NA	2	9	13	4	3	29	0.000409425	6/9	7/3	6/4	7/1
E. coli phage vB_EcoP_G7C	HQ259105	72917	1160	0	NA	5	11	11	2	5	29	0.000397712	6/11	7/3	6/2	7/2
Enterobacter phage EcP1	HQ641380	59080	Not Determined	3	Asn,Pro,Met	0	37	37	0	2	76	0.001286391	8/1	10/1	0	9/1
Enterobacter phage IME11	JX880034	72570	Not Determined	2	Undet, Ile	0	12	8	6	1	27	0.000372055	7/2	6/8	7/2	6/1
Erwinia phage vB_EamP-S6	HQ728266	74669	396	0	NA	0	8	12	0	2	22	0.000294634	7/1	8/1	0	9/2
Pseudomonas phage PA26	JX194238	72321	Not Determined	0	NA	1	5	6	2	2	15	0.000207409	7/1	7/3	7/1	7/1
Pseudomonas phage LIT1	FN422399	72544	632-665	0	NA	1	6	5	2	2	15	0.000206771	7/1	11/1	6/2	7/2
Pseudomonas phage LUZ7	FN422398	74901	632-665	0	NA	0	4	5	2	4	15	0.000200264	7/1	7/1	6/2	7/3
Roseovarius sp. 217 phage 1	FR682616	74583	Not Determined	3	Pro,Ile,Gln	1	9	8	2	2	21	0.000281566	6/9	6/8	7/1	7/1
Roseovarius Plymouth podovirus 1	FR719956	74704	Not Determined	3	Pro,Ile,Gln	1	8	7	2	3	20	0.000267723	6/8	6/7	7/1	7/1
Silcibacter phage DSS3phi2	FJ591093	74611	Not Determined	3	Pro,Met,Ser	1	18	11	6	4	39	0.000522711	9/1	7/2	6/6	6/4
Sulfitobacter phage EE36phi1	FJ591094	73325	Not Determined	3	Pro,Ile,Ser	1	20	12	3	10	45	0.000613706	7/2	7/2	6/3	7/4
Sulfitobacter phage pCB2047-B	HQ317387	74480	Not Determined	13	Met,Tyr,Trp,Leu,Cys,Thr,Pro,Asn,Ile,Lys,Phe,Gln,Arg	0	8	11	4	4	27	0.000362513	6/8	6/11	7/2	7/1
Vibrio phage VBP32	HQ634196	76718	Not Determined	1	Trp	3	11	13	3	1	28	0.000364973	10/1	7/2	9/1	6/1
Vibrio phage VBP47	HQ634194	76705	Not Determined	1	Trp	3	13	12	1	3	29	0.000378072	7/2	10/1	6/1	10/1
V. cholerae phage JA1	JA1	69755	1974	1	Arg	4	19	19	2	3	43	0.000616443	8/1	11/1	6/2	7/1
V. cholerae phage VchO139I	VchO139I	68964	1974	1	Arg	4	18	18	2	3	41	0.000594513	8/1	12/1	6/2	7/1
Lambda	J02459	48502	cos	0	NA	?	40	36	5	2	83	0.00171127	8/2	8/1	7/1	6/2
Mu	AF083977	36717	variable	0	NA	?	30	16	6	3	55	0.001497944	7/4	7/3	6/6	7/1
P1	AF234172	94800	circular	3	Asn,Thr,Met	?	60	71	3	5	139	0.001466245	8/5	8/2	6/3	8/1
T1	AY216660	48836		0	NA	1	35	29	2	0	66	0.001351462	7/6	8/2	7/1	0
T4	AF158101	168903	circular permutation and terminal redundant	8	Gln,Leu,Gly,Pro,Ser,Thr,Met,Arg	15	79	112	0	0	191	0.001130827	8/1	7/2	0	0
T5	AY543070	121750		23	Arg,Ser,Met,Tyr,Glu,Phe,Cys,Asn,Asp,Pro,Lys,Val,Ala,Leu,Gln,Gly,Thr,Ile	?	33	63	0	4	100	0.000821355	6/33	7/1	0	
T7	GU071091	39778		0	NA	?	0	1	0	0	1	2.51395E-05	0	6/1	0	0

Homopolymeric Tracts > 9 nucleotides

Highest number of homopolymeric nucleotides
